# Editorial: *Fiat lux*! Light-driven and light-controlled synthetic biological parts, devices, systems and processes

**DOI:** 10.3389/fbioe.2023.1201962

**Published:** 2023-04-28

**Authors:** Pasquale Stano, Emiliano Altamura, Fabio Mavelli, Vijai Singh, D. Dafydd Jones

**Affiliations:** ^1^ Department of Biological and Environmental Sciences and Technologies (DiSTeBA), University of Salento, Lecce, Italy; ^2^ Department of Chemistry, Università degli Studi di Bari “Aldo Moro”, Bari, Italy; ^3^ Department of Biosciences, School of Science, Indrashil University, Rajpur, Mehsana, India; ^4^ Molecular Biosciences Division, School of Biosciences, Cardiff University, Cardiff, United Kingdom

**Keywords:** optogenetic control, photoswitching, light-inducible processes, light-driven processes, energy transduction

## 1 Research Topic aims and objectives

In the context of synthetic biology, light and other electromagnetic radiation provide a powerful tool to drive and control parts, devices, systems and processes with high target specificity and switching efficiency. Thanks to its peculiar features in terms of wavelength and energy, light allows spatio-temporal control of molecular and supramolecular systems that ultimately lead to (bio)chemical and (bio)logical behavior—such as optogenetic control and energy fueling.

In the recent years, a large number of studies have been devoted to the design and construction of molecular systems, either *in vitro* or *in situ/in vivo*, that convincingly show the versatility and the power of such approaches. Examples involve both visible and near-infrared light in bacteria, yeast, mammalian and plant cells. The light control of gene expression has been used for triggering processes such as signaling, recombination, initiation of translation, production of chemicals and peptides, apoptosis, intracellular transport, and cell differentiation. Additional cases refer to protein localization distribution, protein degradation, protein homo- and hetero-dimerization, alteration of a protein’s metal binding behaviour, and also protein coacervation. Complex processes including biofilm formation, cell differentiation and morphogenesis have been considered.

All these achievements excitingly impact current synthetic biology research because of the intrinsic versatility and power of light, which makes it easier to drive and control a large number and variety of processes.

The Research Topic “*Fiat Lux! Light-Driven and Light-Controlled Synthetic Biological Parts, Devices, Systems and Processes*”, which was focused on the above-mentioned approaches, collects seven articles (five reviews, one perspective article, one original research paper) that deals with the subject of light-driven and light-controlled synthetic systems and processes, in particular optogenetics, but also related approaches. The resulting picture provides a cross section of the state of the art in the field, and can inspire and guide further investigations.

## 2 Highlights from the Research Topic articles

The Research Topic “*Fiat Lux! Light-Driven and Light-Controlled Synthetic Biological Parts, Devices, Systems and Processes*” counts seven articles. The five reviews and the perspective paper can be advantageously utilized as a Research Topic of discussions about relevant issues in light-controlled biodevices: from their functioning to the investigation platform, from their design to applications.

Two comprehensive reviews must be mentioned firstly. Ohlendorf and Möglich review the fundamentals, the advances, and the perspectives of optogenetically regulated gene expression in bacteria. By highlighting three fundamental strategies, namely, light-sensitive two-component systems, oligomerization, and second-messenger signaling, the authors highlight relevant application areas of optogenetic control. One is the enhanced yields that can be achieved in microbial production processes. Next, the control of the secretion of compounds that grant health benefits to the animal host by light-responsive bacteria which reside within the bodies of animals. Third, optogenetics can lead to the synthesis of precisely structured, novel biomaterials.

Baumschlager, on the other hand, presented a review that discusses important aspects for engineering of light-controllable proteins through selected examples. The focus is on non-neuronal optogenetics, chromophore availability, general strategies for creating light-controllable functions, modification of the photosensitive domains and their fusion to effector domains, as well as tuning concepts for opto-proteins.

The next two articles deal, instead, with more specific research topics. In particular, Dwijayanti et al., focused their review on the progress made on systems with multiple photoreceptors, each sensing its dedicated wavelength. The combination of multiple photoreceptors allows a coordination of cellular responses. Recent works and challenges on multiplexed optogenetic circuits in natural and engineered systems are discussed.

The contribution written by Zhanget al., instead, is a perspective article on light switchable two-component protein dimerization systems. The study provides categories for mechanisms and design approaches of these dimerization systems, which have been recently progressed by the discovery of photoreceptor-based interaction systems, by the engineering of light-actuatable binder proteins, and by the development of photoactivatable compounds as dimerization inducers.

The methodologies and experimental platform for studying optogenetic controls have been thoroughly discussed by Kumar and Khammash, who reviewed the evolution of light-induction hardware-software platforms from simple illumination set-ups to sophisticated microscopy, microtiter plate and bioreactor designs, and discuss their respective advantages and disadvantages. Moreover, experimental approaches such as treatments of different cell types and culture volumes, with induction capabilities ranging from single cell stimulation to entire cell culture illumination, automated measurement and stimulation schemes on these platforms.

On the other hand, Månsson et al. illustrated, in another review, mechanisms and applications of “OptoGels”, i.e., hydrogels with light-programmable properties endowed by photoswitchable proteins (“optoproteins”) found in nature. Thanks to conjugation chemistry OptoGels with a combinatorially large design space (still not well explored) can be designed, resulting in a rich variety of tunable material properties. The potential future applications of OptoGels range from mechanobiology to 3D cell and organoid engineering, as well as programmable cell eluting materials.

An original research article (the only one in the Research Topic) has been presented by Hilgers et al. Their investigation focuses on photocaged inducer molecules (e.g., photocaged isopropyl-β-D-1-thiogalactopyranoside, cIPTG) as well-established optochemical tools for regulating bacteria gene expression by light. The study is based on the photoactivation of gene expression in *Rhodobacter capsulatus* by using different cIPTG variants, under phototrophic and non-phototrophic cultivation conditions. The authors have identified a promising compound, 6-nitropiperonyl-(NP)-cIPTG. The optochemical approach was then successfully applied to the induction of carotenoid biosynthesis.

Finally, Albanese et al. have reported about the latest strategies for the assembly of energetically autonomous “bottom-up” artificial cells. The authors refer to the branch of synthetic biology devoted to the construction of cell-like systems able to mimic fundamental aspects of living cells, yet being minimally complex. The review focuses on the exploitable (and exploited) bio-inspired mechanisms of light transduction aimed at supporting internalized metabolic pathways in artificial cells. The concepts behind the construction of mono- and multi-compartment artificial cells capable of light-driven proton gradient and ATP production are systematically presented and discussed in the context of next-generation artificial cells.

## 3 Concluding remarks

The Research Topic of the above-mentioned papers is of great interest for synthetic biologists who intend to learn about the state-of-the-art in using light to control biological processes. The articles collectively offer a wide perspective on a variety of approaches and investigations carried in the past years on optogenetic control and related systems and on the bottom-up construction of cell-like systems that exploit light as ultimate energy source.

